# Effect of Au nanoparticles on ZnO nanorods/α-Fe_2_O_3_ electrochemical sensor performance

**DOI:** 10.1039/d5ra05210f

**Published:** 2025-09-17

**Authors:** Sreymean Ngok, Xianjie Liu, Magnus Willander, Omer Nur

**Affiliations:** a Physics Electronics and Mathematics, Department of Science and Technology, Linköping University SE-601 74 Norrköping Sweden sreymean.ngok@liu.se +46 11 363219; b Department of Science and Technology, Laboratory of Organic Electronics, Linköping University Norrköping SE-601 74 Sweden

## Abstract

In this study, the development of an efficient nano-electrode for fabricating electrochemical sensors to detect arsenic(v) in drinking water is presented. This nano-electrode is composed of a metal–semiconductor hybrid, namely ZnO NRs/α-Fe_2_O_3_/Au NPs, denoted as (ZFA), which was synthesized in three separate steps, including the low-temperature hydrothermal and dip-coating methods. The properties of the nanocomposite were characterized by UV-vis, FESEM, XRD and XPS. Moreover, its electrochemical characteristics were analyzed *via* several techniques, such as linear sweep voltammetry and square wave voltammetry in different water solutions with a wide range of arsenic concentrations from 0 to 50 μg L^−1^. Results indicate that the ZFA nanocomposites that were prepared with different concentrations of Au NPs showed different characteristics. From optical measurements, the best Au NP sample having the highest surface plasmon resonance (SPR) effect was determined, and this sample was further utilized for sensing. Moreover, using the optimized sample and from electrochemical studies, the arsenic(v) sensor's limit of detection was found to be 2.25 ppb, which is lower than the maximum dose recommended by the World Health Organization. In general, the results indicate that the addition of Au NPs led to better optical absorption properties. The findings of this study indicate that the addition of Au improves the electrochemical catalytic activity of the ZFA nanocomposite, which can be utilized as an electrode to further develop efficient arsenic(v) sensing systems for detection in drinking water.

## Abstract 2

In this study, the development of an efficient nano-electrode for fabricating electrochemical sensors to detect arsenic(v) in drinking water is presented.

## Graphical abstract

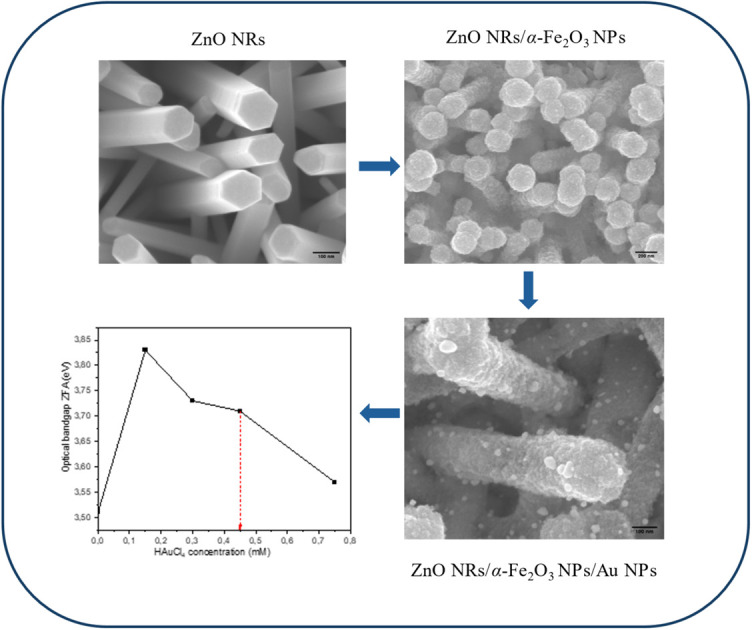

## 1. Introduction

In recent years, arsenic detection has attracted much attention.^1,2^ Arsenic is a toxic element that usually contaminates drinking water.^3^ In the earth's crust, it exists in four oxidation states, −3, 0, +3, and +5, where the +3 and +5 oxidation states are the toxic forms found in natural groundwater resources.^4^ The World Health Organization has fixed guideline values of 50–10 ppb for the maximum allowed contaminant level of arsenic in drinking water.^5^ Electrochemical sensing is a promising method that can provide a low-cost and convenient solution for the detection of variable analytes and is widely utilized in different applications.^6^ Zinc oxide (ZnO) nanostructures have intrinsic n-type semiconducting polarity, a direct wide bandgap (3.37 eV), interesting structural and mechanical properties, with a variety of synthesis methods and exist in many morphologies. This makes ZnO a suitable choice for many applications, such as photodetectors,^7^ field-effect transistors,^8^ piezoelectric nanogenerators^9^ and electrochemical sensors.^10^ By contrast, hematite (α-Fe_2_O_3_) has a direct wide bandgap (2.2 eV) and is widely used in catalysis, pigments and different sensor applications.^11–14^ Although bare nanostructures have improved the performance and utilization of materials for many applications, by combining different nanomaterials into a synergetic design, much more can be gained. By compositing different nanomaterials with the proper design of the conduction and valence band offsets, efficient separation of carriers can be achieved. ZnO/α-Fe_2_O_3_ nanocomposites have been proposed and utilized for gas sensing.^15^ Nevertheless, the performance of ZnO/α-Fe_2_O_3_ nano-composites in sensing applications has been limited due to their low electrical conductivity.^16,17^ In order to improve the performance of ZnO/α-Fe_2_O_3_ nanostructures as electrochemical sensors, electrode decoration with gold nanoparticles (Au NPs) can lead to higher electrical conductivity,^18^ which is particularly promising.^19–21^ Additionally, Au NPs increase electrical conductivity when agglomeration occurs by overlapping electron wave functions of the clustered nanoparticles and by electron tunneling between discrete nanoparticles; this also leads to a relatively faster electron transport and efficient charge transfer.^22,23^ Gold nanoparticles (Au NPs) constitute an excellent plasmonic metal with the potential for developing efficient devices due to its excellent properties, *e.g.*, catalytic performance, biocompatibility, *etc.*^24–26^

In this paper, Au NPs were deposited onto the surface of a ZnO NRs/α-Fe_2_O_3_ NPs nanocomposite, denoted as (ZFA), and grown on FTO substrates for efficient arsenic(v) sensing in drinking water. The ZFA nanocomposite electrodes were grown by optimized hydrothermal methods, followed by the dip coating technique. Different analytical, structural, and optical characterization tools have been utilized to investigate the different properties of the grown material. The sensor performance was studied using a variety of electrochemical techniques. The detection performance of the best ZFA nanocomposite electrode showed promising results and can be further developed as an excellent candidate for the detection of arsenic(v) in drinking water.

## 2. Experimental

### 2.1 Materials

Zinc nitrate hexahydrate (Zn(NO_3_)_2_·6H_2_O), hexamethylenetetramine (HMT), iron(iii) nitrate nonahydrate (Fe(NO_3_)_3_·9H_2_O), HAuCl_4_, and arsenic standard solution were purchased from Sigma Aldrich. All the chemicals used here were of analytical grade and were used without further purification.

### 2.2 Preparation of ZnO NRs

The low-temperature hydrothermal chemical method was utilized to synthesize ZnO nanorods (NRs).^27^ Firstly, the FTO substrate was cut into sizes of approximately 10 mm × 15 mm. These FTO substrates were cleaned separately with deionized water (DI), acetone, and isopropanol in an ultrasonic bath for 10 minutes each, and then they were dried under a nitrogen flow. These substrates were then spin-coated with ZnO nanoparticles to act as seeding sites. To ensure reasonable surface coverage, this process was repeated three times at 3000 rpm. This was followed by annealing at 120 °C for 25 min. Zinc acetate dihydrate was dissolved in methanol to form a 0.01 M solution and then subjected to vigorous stirring at about 60 °C. A 0.03 M solution of KOH in methanol was added dropwise to the 0.01 M solution above. To get the desired rod-shaped nanoparticles, the mixture was subjected to two stirring processes. First, it was stirred for 2 h at 60 °C, and in the second stirring process, it was stirred for 12 h at room temperature.^28^ The precursor solution to synthesize the ZnO NRs was prepared by dissolving an equal molarity (0.05 M) of Zn(NO_3_)_2_·6H_2_O and HMT in 100 mL of DI water. The FTO substrates were then hung, having the prepared surface pointing downwards, and then dipped into the growth solution. The beaker was shielded and inserted into a laboratory oven kept at 95 °C for a duration of 5 hours. After completion of the growth duration, the samples were cleaned with DI water and blown dry by flowing nitrogen.

### 2.3 Preparation of ZnO NRs/α-Fe_2_O_3_ nanoparticles

In the second step, α-Fe_2_O_3_ nanoparticles (NPs) were synthesized by the dip-coating method.^17^ To deposit α-Fe_2_O_3_ NPs onto the surface of the ZnO NRs, a dipping process was used. The as-grown ZnO NRs were dipped into a solution of 0.06 g Fe(NO_3_)_3_·9H_2_O dissolved in 20 mL of DI water. This process was applied for 2 minutes, and it was repeated three times to obtain a uniform spatial coverage over the ZnO NRs surface. After completing the previous process, nitrogen was blown over the samples at room temperature. To convert the hydroxyl-containing phase of the Fe_2_O_3_ to the pure α-phase, the samples were annealed at 400 °C for 2 hours in an air environment using a conventional laboratory hot plate. The ZnO NRs/α-Fe_2_O_3_ NPs electrode is denoted as (ZF).

### 2.4 Preparation of the ZnO NRs/α-Fe_2_O_3_ NPs/Au NPs nanocomposite

**Fig. 1:**
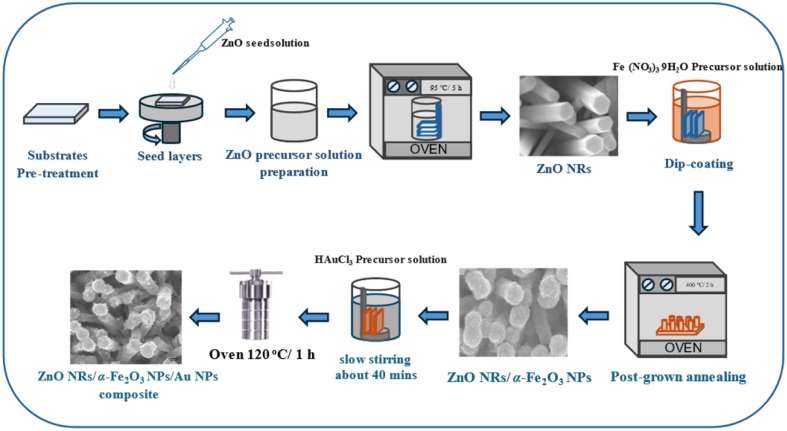
Schematic displaying the synthesis of the ZFA nanocomposite electrodes.

Au NPs were synthesized by a hydrothermal method.^29^ The modification of Au NPs onto the ZnO NRs/α-Fe_2_O_3_ NPs was achieved using precursor solutions of different concentrations (0.15–0.75 mM HAuCl_4_); an aqueous solution was prepared using 1 mL of methanol, then it was added to 20 mL of DI water. The samples are denoted (ZFA-0.15 mM), (ZFA-0.3 mM), (ZFA-0.45 mM), and (ZFA-0.75 mM), respectively. Using a 0.01 M sodium hydroxide (NaOH) solution, the pH of the solution was adjusted to 8. The ZF-grown nanocomposites were inserted into the above solution in an autoclave stainless steel vessel. Then, the hydrothermal synthesis of Au NPs deposited on the surface of ZF samples was carried out in a conventional oven kept at 120 °C for 1 hour. The samples were left to cool down to room temperature. The ZFA nanocomposite electrodes were cleaned using DI water, followed by a drying process using flowing nitrogen. Finally, the samples were annealed at 80 °C under a vacuum environment. Fig. 1 displays a schematic diagram of the synthesis of the ZFA nanocomposite electrodes.

### 2.5 General characterization techniques

The morphological and structural properties of all grown samples were characterized by scanning electron microscopy (FESEM, Sigma 500 Gemini) and powder X-ray diffraction (Philips's powder diffractometer), respectively. To gain insight into the optical properties, a UV-vis spectrophotometer (PerkinElmer Lambda 900) was used. The chemical composition and the oxidation states were monitored by X-ray photoelectron spectroscopy.

### 2.6 Electrochemical characterization

An Autolab potentiostat (Metrohm) was used to perform electrochemical measurements. These electrochemical measurements were investigated using a three-electrode configuration. In this three-electrode configuration, Ag/AgCl (3 M KCl) was used as the reference electrode, platinum wire was the counter electrode, and the nanocomposite electrode was the working electrode. Here, the areas of all working electrodes (ZNRs, ZF, and ZFA) were the same, around 0.3 cm^2^.

## 3. Results and discussion

### 3.1 Morphological analysis

**Fig. 2:**
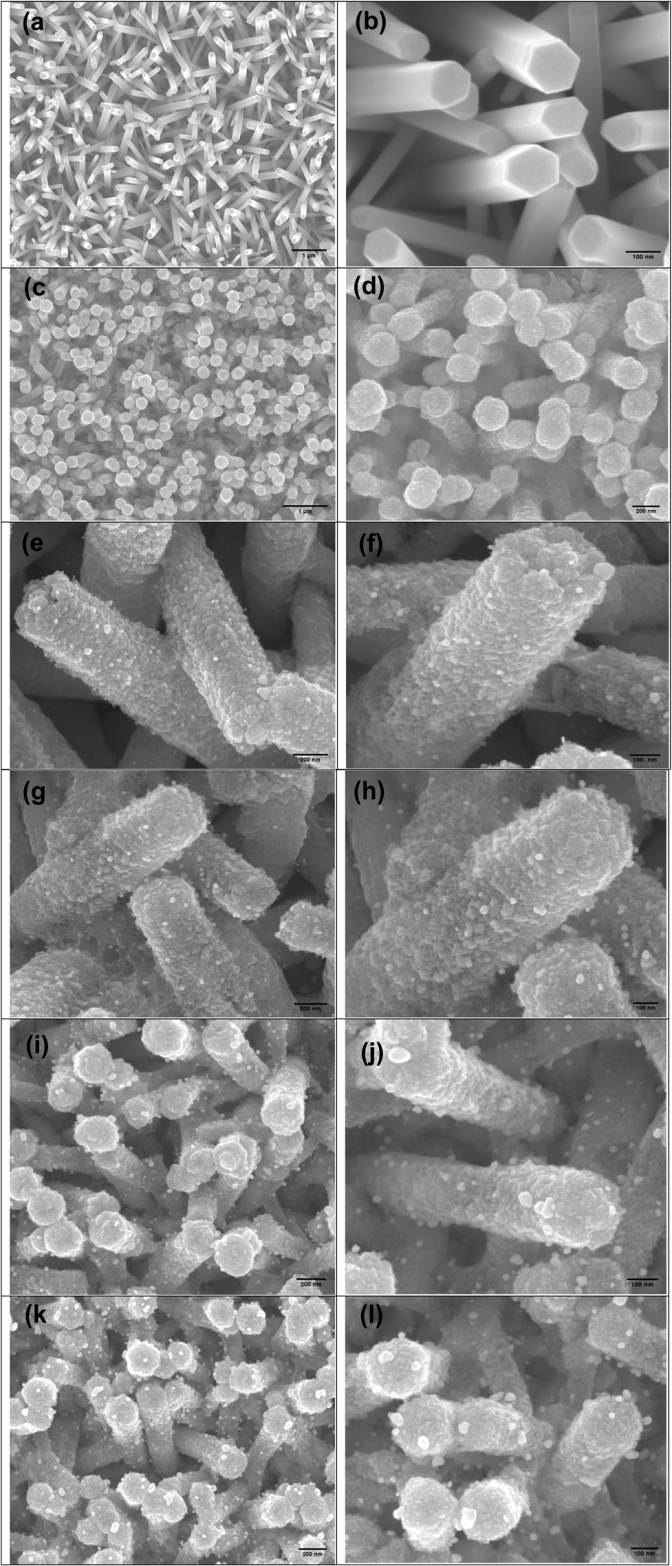
SEM images of (a and b) ZnO NRs, (c and d) ZnO NRs/α-Fe_2_O_3_ NPs, and ZnO NRs/α-Fe_2_O_3_ NPs/Au NPs with different concentrations of HAuCl_4_: (e and f) 0.15 mM, (g and h) 0.3 mM, (i and j) 0.45 mM, (k and l) 0.75 mM.

The morphologies of the ZNRs, ZF, and ZFA nanocomposites were studied using FESEM, as demonstrated in Fig. 2. The morphology of pure ZNRs shows the expected hexagonal shape of the ZnO nanorods; these nanorods are clear, dense, and relatively vertically aligned to the FTO surface, as shown in Fig. 2a and b. Fig. 2c and d show that the α-Fe_2_O_3_ NPs grew on the surface of the ZNRs, which have much larger particle sizes. Moreover, the particles of the α-Fe_2_O_3_ were observed to cover the ZNR surface, forming a core–shell configuration. To investigate and optimize the molar concentration of the HAuCl_4_, which is the precursor for depositing Au NPs on the surface of the ZF nanocomposites, various concentrations were used. Fig. 2e–l demonstrates the FESEM images of Au NPs modifying the surface of the ZF after the hydrothermal process in 0.15, 0.3, 0.45, and 0.75 mM of HAuCl_4_, respectively. For all sample preparations, the hydrothermal reaction time was fixed at 1 h. We observed that the spatial coverage of the Au NPs grown on the ZF surface became denser as the concentrations of the HAuCl_4_ increased. A very dense Au NPs covering of the surface of the working electrode was observed when the gold precursor concentration was as high as 0.75 mM. ZFA is shown in Fig. 2k and l (see XRD discussion below). These SEM images revealed that, in general, all the ZF nanocomposites were modified by Au NPs with a size of approximately 10–50 nm with different spatial coverage, depending on the Au precursor concentration (see the related discussion in the UV-visible section). When the concentration of the HAuCl_4_ was 0.45 mM, Au NPs (smaller Au NPs compared to the case with 0.75 mM) were attached uniformly to the surface of ZF, as shown in Fig. 2i and j. As the concentration of HAuCl_4_ decreased, a lower density of Au NPs was observed on the surface of ZF (Fig. 2g and h). When the concentration of the Au precursor was 0.15 mM, almost no Au NPs were formed on the surface of the ZF (Fig. 2e and f). The SEM images shown in Fig. 2e–l suggest that the density of Au NPs on the surface of ZF could be dependent on the concentration of HAuCl_4_.

### 3.2 Optical properties

**Fig. 3:**
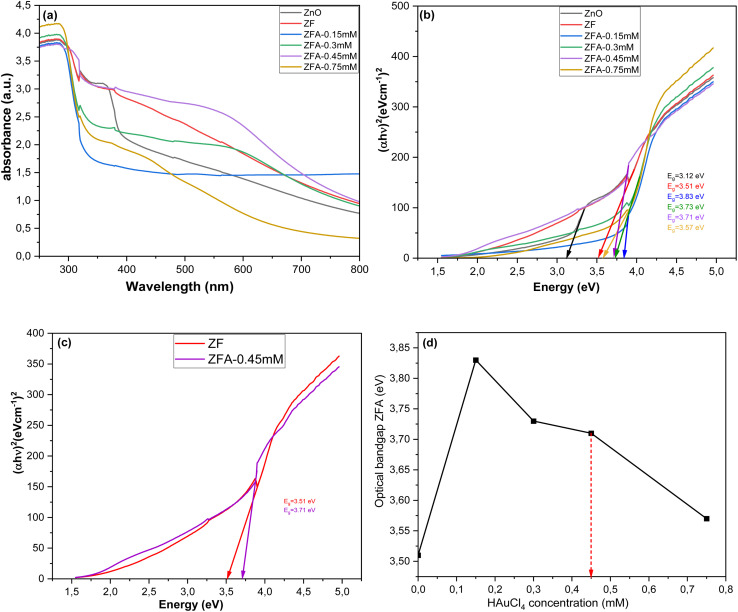
UV-vis absorption spectra of (a) ZnO NRs, ZnO NRs/α-Fe_2_O_3_ NPs and ZnO NRs/α-Fe_2_O_3_ NPs/Au NPs composite with different concentrations of HAuCl_4_. (b) Evaluation of the bandgap energies of ZnO NRs, ZnO NRs/α-Fe_2_O_3_ NPs, ZFA-0.15 mM, ZFA-0.3 mM, ZFA-0.45 mM and ZFA-0.75 mM. (c) Evaluation of the bandgap energies of ZnO NRs/α-Fe_2_O_3_ NPs and ZFA-0.45 mM. (d) The synergetic optical bandgap energies as a function of the Au precursor concentration, *i.e.* HAuCl_4_ concentration.

All the grown nanocomposite samples, *i.e.*, ZNRs, ZF, and ZFA nanocomposites, were investigated using UV-visible spectroscopy. These optical characterizations are shown in Fig. 3. The absorption peak at about 397 nm (*E*_g_ = 3.12 eV) is assigned to the intrinsic exciton absorption of the ZNRs, which is in good agreement with the XRD results. After the ZNRs were modified by the α-Fe_2_O_3_ NPs as shells, and due to the deposition of the Au NPs on the surface of the ZF samples, the absorption range was observed to extend into the visible region. The absorbance intensity was also observed to increase in the visible light region. The absorption peak also increased due to the surface plasmon resonance (SPR) absorption of Au NPs deposited on top of the ZF. The energy bandgaps were found to be 3.83 eV, 3.73 eV, 3.71 eV, and 3.57 eV for the ZFA-0.15 mM, ZFA-0.3 mM, ZFA-0.45 mM, and ZFA-0.75 mM, respectively. The absorption spectra of the SPR band due to the Au NPs in the wavelength range of 500–600 nm for the ZFA nanocomposite samples were also recorded and can be seen in Fig. 3a. There was an increase in the absorption between 500–600 nm, especially for the ZFA-0.45 mM sample. For the other three Au NPs samples, the SPR effect was observed to be weaker (ZFA-0.15 mM, ZFA-0.3 mM, and ZFA-0.75 mM). The observed increased absorption for the ZFA-0.45 mM is also consistent with the XRD and the electrical sensing experiment results (see below). The absorption band between 500–600 nm, which corresponds to the SPR effect of Au, confirms that Au NPs were successfully deposited on the surface of the ZF nanocomposite.^30–33^ To estimate the energy band gap of each sample, Tauc plots were used to extract the optical bandgap, as shown in Fig. 3b.^34^ As the amount of Au NPs increased, the optical band gap was observed to decrease due to the enhancement of the carrier species in the valence band and conduction band.^35^ Additionally, Au NPs were introduced into the ZF solution, which could be the cause of the decrease in the band gap energy due to unloaded Au NPs in the solution.^36^Fig. 3c shows the Tauc plots for the ZF reference and the ZFA-0.45 mM samples for comparison. Fig. 3d shows the optical bandgaps of all the ZFA samples. The synergetic optical bandgap decreases with the increasing Au precursor concentration. The red arrow indicates the band gap of the ZFA-0.45 mM sample, which showed the highest optical absorption due to the SPR, providing the best sensing performance, as discussed below.

### 3.3 XRD analysis

**Fig. 4:**
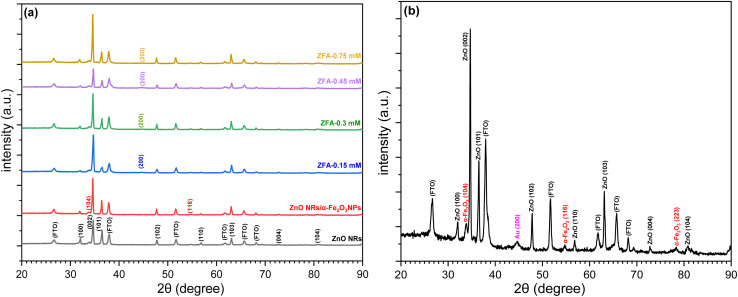
XRD patterns of (a) ZnO NRs, ZnO NRs/α-Fe_2_O_3_ NPs with different concentrations of Au NPs (b) ZFA-0.45 mM.

The crystalline nature of the as-prepared ZNRs, ZF, and ZFA nanocomposite electrodes was investigated using XRD, as shown in Fig. 4. The XRD pattern of the ZNRs on FTO substrates are shown in Fig. 4a, in which the 2*θ* diffraction peaks were well matched with the standard JCPDS no.: 00-036-1451 card, and the diffraction pattern of ZF in which 2*θ* diffraction peaks at 33.64° and 54.46° correspond to the (104) and (116) reflection planes of the rhombohedral structure of α-Fe_2_O_3_ (JCPDS: 00-033-0664), respectively. The diffraction peak at 44.6° is attributed to the (200) plane of Au NPs, as shown in Fig. 4b. Because the ZF masks the Au NPs peaks, no more Au NPs peaks were detected from the ZFA nanocomposite electrode samples. The results indicate that the ZFA nanocomposite has been produced successfully. On investigating the Au peak by varying the Au precursor and comparing the XRD results with the SEM morphology seen in Fig. 2 for all the samples with Au NPs, it is evident that there is a consistency, as discussed below. The XRD spectra of the Au peak (Fig. S1 in the SI) indicate that the Au peak is only observed for the 0.45 mM and the 0.75 mM concentrations. Nevertheless, the UV-visible absorption spectra results showed that the 0.45 mM sample possesses a higher SPR absorption peak (between 500–600 nm). Even the SPR absorption peak for the ZFA-0.75 mM is lower than the corresponding absorption peaks for the ZFA-0.15 mM and ZFA-0.3 mM. To correlate these observations with the morphology and spatial distribution of the Au NPs, a comparison of Fig. 2i–l was made. On comparing the 0.45 mM and 0.75 mM concentrations of the HAuCl_4_ samples, the SEM indicates that on reaching 0.75 mM, the spatial distribution of the Au NPs had increased. This increase is even evident from the presence of more Au NPs, which are even seen on the top of the polar surface of the NRs (Fig. 2k and l). The crystallite size (*D*) of Au particles is calculated from the well-known Debye–Scherrer formula:^37^
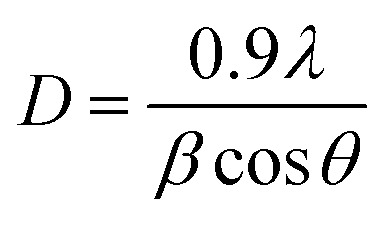
where *λ* is the wavelength of monochromatic Cu-Kα radiation (1.54056 Å), *θ* is Bragg's angle related to the (200) planes of Au NPs, whereas *β* denotes the Full Width at Half Maxima (FWHM) of the broadened and intense peaks. The average particle size of Au NPs was calculated and was found to be in the range of 10–30 nm.

### 3.4 XPS analysis

**Fig. 5:**
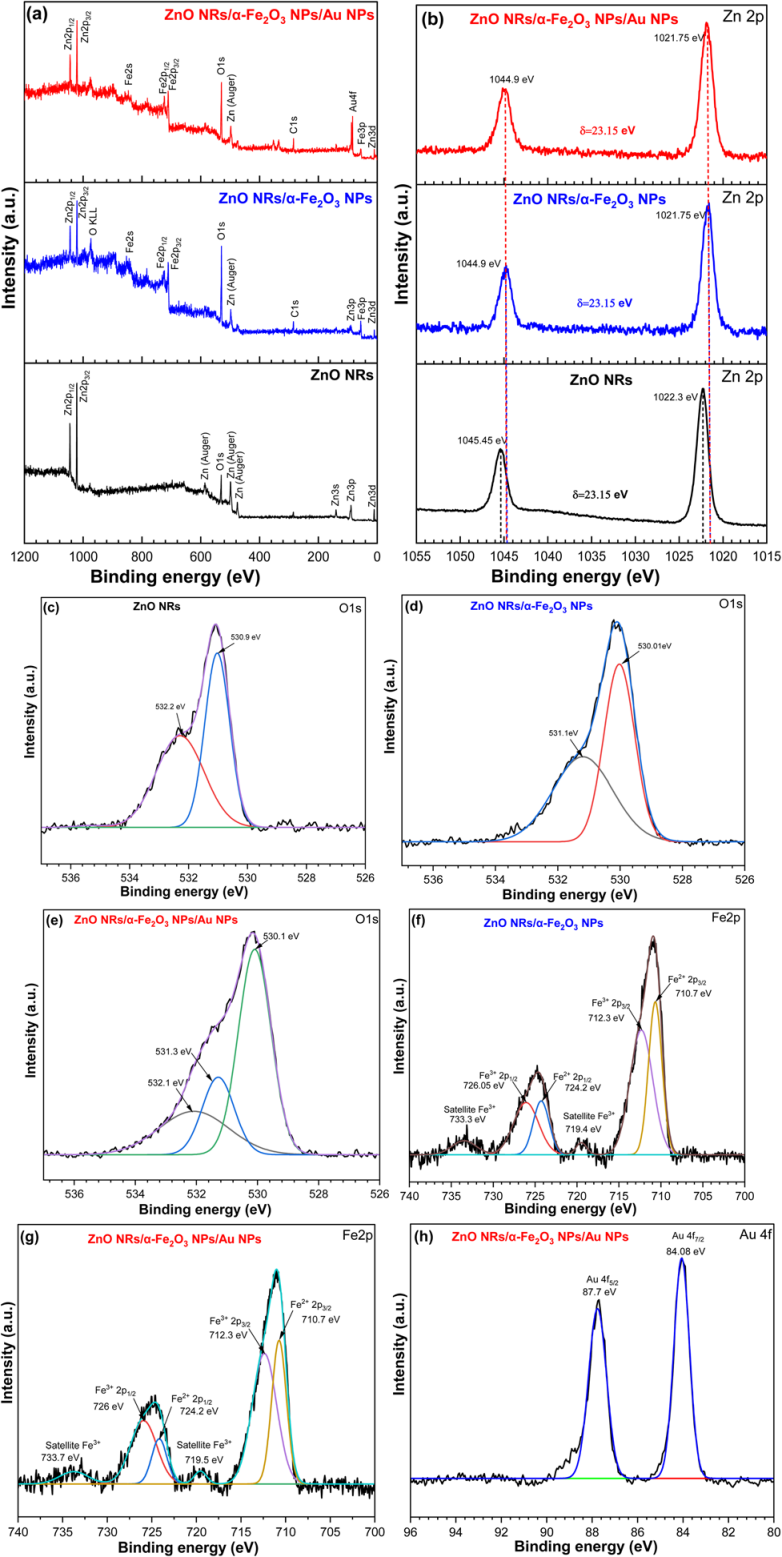
XPS analysis. (a) The survey scan spectra of ZnONRs, ZnONRs/α-Fe_2_O_3_ NPs and ZnONRs/α-Fe_2_O_3_NPs/AuNPs. The high-resolution spectra of ZnONRs, ZnONRs/α-Fe_2_O_3_ NPs and ZnONRs/α-Fe_2_O_3_NPs/AuNPs: (b) Zn 2p, (c–e) O 1s, (f and g) Fe 2p and (h) Au 4f.

The chemical composition and oxidation states for all the grown samples, including the ZNRs, ZF, and ZFA, were determined by X-ray photoelectron spectroscopy (XPS) measurements. The results were fitted by a Gaussian fitting method as shown in Fig. 5. In Fig. 5a, long survey scan spectra of the bare ZNRs, ZF, and ZFA are shown. They confirmed the presence of Zn 3d, 3p, 3s, 2p, auger, O 1s, Fe 2p, and Au 4f. After the modification with the α-Fe_2_O_3_ NPs on the ZNRs, the spectrum showed some extra peaks of Fe 3p, 2p, 2s, and O KLL.^38,39^ A high-resolution spectrum of the Zn 2p is shown in Fig. 5b, where a doublet peak with binding energies of 1022.3 eV and 1045.45 eV is observed. These are contributions from 2p_3/2_ and 2p_1/2_ of Zn with a spin–orbit splitting energy of 23.15 eV. This proves the presence of Zn^2+^.^40–42^ If we compare the high-resolution spectrum of Zn 2p of pure ZnO NRs to that of the ZF and ZFA peaks, it is evident that the Zn 2p peak is shifted to higher binding energy. The core-level XPS spectra of O 1s of the pure ZNRs, ZF, and ZFA samples are shown in Fig. 5c–e with peak binding energies at 530.9, 530.01, and 530.1 eV, corresponding to the O of ZnO,^43,44^ whereas the peaks at 531.1 eV and 531.3 eV are ascribed to the oxygen vacancies. The peak at 532.1 eV results from the adsorbed oxygen species on the surface of the material.^45^ The core-level peaks of the Fe 2p region include Fe^2+^ 2p_3/2_, Fe^3+^ 2p_3/2_, Fe^2+^ 2p_1/2_, and Fe^3+^ 2p_1/2_ peaks at 710.7 eV, 712.3 eV, 724.2 eV, and 726 eV, respectively, as displayed in Fig. 5f and g, indicating the presence of Fe^2+^ and Fe^3+^ species.^38,46^ Additionally, two satellite peaks were observed at 719.5 eV and 733.7 eV.^47,48^ In Fig. 5h, Au 4f_7/2_ and Au 4f_5/2_ XPS peaks at 84.08 eV and 87.7 eV were observed, corresponding to elemental Au.^49,50^ Therefore, from these XPS measurements, it was concluded that Au NPs were successfully deposited on the ZF.

### 3.5 Electrochemical characterization

**Fig. 6:**
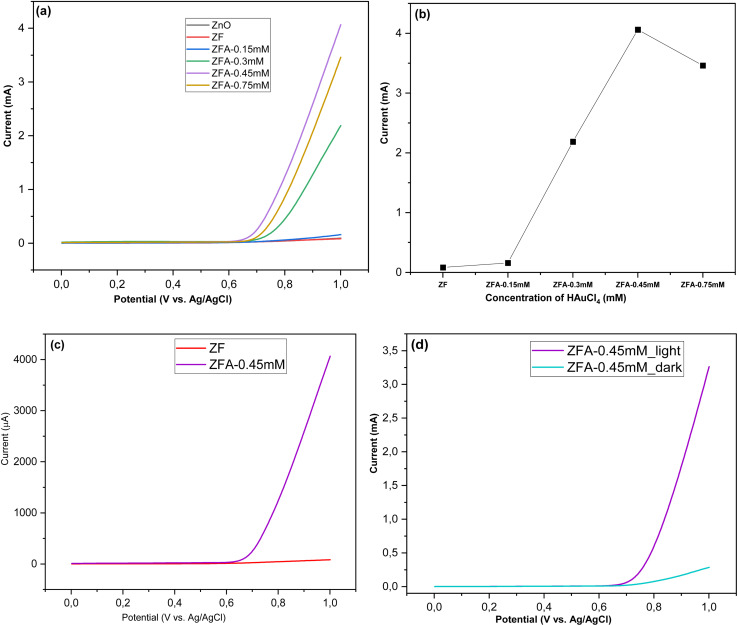
(a) LSV responses of the ZnO NRs, ZnO NRs/α-Fe_2_O_3_ NPs and different concentrations of the Au NP electrode. (b) The corresponding linear calibration plots of the net current *versus* the Au NPs precursor. (c) LSV responses of ZF and ZFA-0.45 mM. (d) LSV responses of ZFA-0.45 mM under light and dark conditions.

**Fig. 7:**
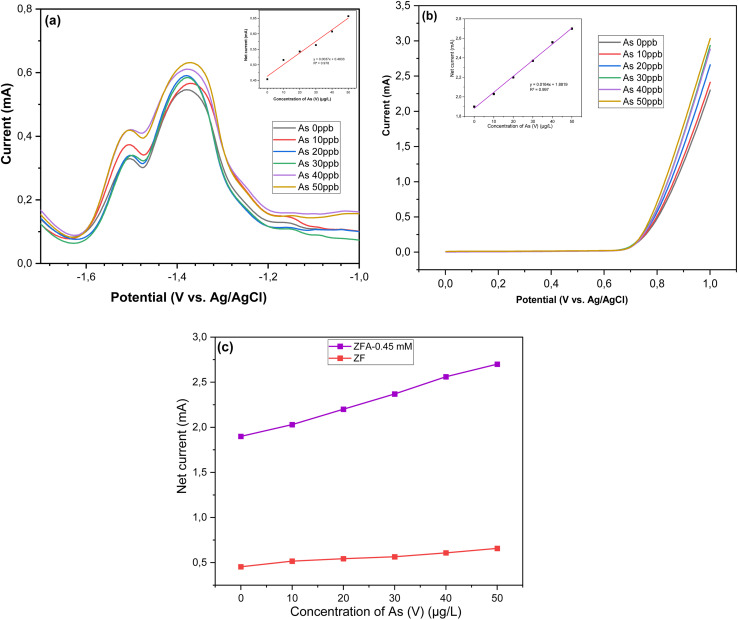
(a) SWV responses of the ZnO NRs/α-Fe_2_O_3_ NP electrode towards arsenic(v) for the concentration range of 0–50 μg L^−1^, and the corresponding linear calibration plots of the net current *versus* arsenic(v) concentrations. (b) LSV responses of the ZFA-0.45 mM electrode towards arsenic(v) in a concentration range from 0 to 50 μg L^−1^, and the corresponding linear calibration plots of the net current *versus* arsenic(v) concentrations. (c) A comparison of the corresponding linear calibration plots of the net current *versus* arsenic(v) concentrations for ZF and ZFA-0.45 mM.

Linear sweep voltammetry (LSV) measurements of the bare ZNRs, ZF, and ZFA nanocomposites were performed to investigate the electrochemical behavior of these nanocomposite electrodes by using 70 mL of 1 M KOH and arsenic(v) solution having different concentrations. The LSV recorded potential windows, ranging from 0 to 1 V *vs.* Ag/AgCl at a scan rate of 100 mV s^−1^ in 1 M KOH electrolyte, are shown in Fig. 6a. The LSV profiles with 1 M KOH solution for the bare ZNRs, ZF, and ZFA nanocomposites, which were grown with different concentrations of HAuCl_4_, show that the peak current is higher for nanocomposites containing Au NPs. Fig. 6a shows that the current increases on increasing the Au precursor concentration up to 0.45 mM; a drop in the current is observed on reaching the concentration of 0.75 mM. This implies that the optimum concentration of the Au precursor is 0.45 mM. The highest current was observed for the ZFA nanocomposite with 0.45 mM, consistent with the UV-visible results (Fig. 2), due to SPR, as the increase was observed in the wavelength range of 500–600 nm. The sample with 0.45 mM concentration (ZFA-045 mM) was then used for sensing experiments. To further study the SPR effect, the current was measured under dark conditions and with light on. As shown in Fig. 6d, the current difference between the light on and off environments at the same voltage for the ZFA-0.45 mM nanocomposite electrode indicates that SPR and, probably, hot electrons are contributing to the improvement of the current. On measuring and comparing the current for the bare ZF and the ZFA-0.45 mM, an increase in the stripping current was observed due to the presence of the Au NPs (Fig. 6c). Also, the ZFA-0.45 mM was found to have stronger absorption (see the UV-visible spectra in Fig. 2), leading to a better detection performance toward arsenic(v) in drinking water. Fig. 7a shows the square wave voltammetry (SWV) responses of the ZF; when the arsenic concentration increased, it was observed that the stripping current had increased. The calibration plot was found to be linear over a concentration range of 0 to 50 μg L^−1^ of arsenic(v), with a regression equation extracted from the calibration curve given by *y* = 0.0037*x* + 0.4633 (*R*^2^ = 0.978) as displayed in Fig. 7a. These findings show that the detection of arsenic(v) in an aqueous solution was efficiently achieved, even at a very low concentration of arsenic(v). The lower limit of detection (LOD) was further determined by using the equation of LOD = 3*σ*/*S*, where *σ* is the standard deviation of the calibration curve that contains samples from different measurement ranges (*n* = 3), and *S* is the slope of the calibration curve.^51^ The LOD was calculated to be 6.89 ppb, which is lower than the maximum allowed value of 10 ppb suggested by the World Health Organization (WHO).^2,52^Table 1 displays the LOD of previous similar experiments for the detection of arsenic in drinking water. Further, after the composition of Au NPs using 0.45 mM concentration of HAuCl_4_, the ZFA-0.45 mM electrode was used for the detection of arsenic(v) with concentration in the range 0–50 μg L^−1^ (ppb), and it indicated that the peak current increases with increasing the arsenic(v) concentration in the electrolyte as demonstrated in Fig. 7b. When investigating the calibration plot, we observed that it was linear over a wide concentration range of 0–50 ppb of arsenic(v). The extracted regression equation was found to be *y* = 0.0164*x* + 1.8819 (*R*^2^ = 0.997) as shown in Fig. 7b. The LOD value of the ZFA-0.45 mM electrode was found to be 2.25 ppb, which is also comparable to those reported previously, as seen in Table 1. In Fig. 7c, a comparison of the corresponding linear calibration plots of the net current against arsenic(v) concentrations demonstrated that the current of the ZFA-0.45 mM is much higher than for the bare ZF. The ZFA-0.45 mM electrode performance revealed that the presence of Au NPs on the ZF surface led to better sensitivity. Some previous similar investigations reported higher sensitivity with lower detection limits, but drawbacks, like cost, the complicated fabrication protocol, and the use of toxic materials, need to be solved. The stability of the ZFA-0.45 mM electrode was investigated, and the LSV measurement was repeated in 50 μg L^−1^ of arsenic(v) solution under optimal conditions. The value of the relative standard deviation (RSD) was found to be 9.43%. This value confirms the stability of the proposed sensor for use up to 4 times for detection purposes without significant changes in the result.

**Table 1:** Some previously published arsenic detection results

Materials	Detection method	Arsenic concentration	LOD	Ref.
ZnO quantum dots		10–100 ppb (fluorescent)	28 ppb	53
ZnO–GO nanocomposite	DPV	80 μM	0.24 μM	54
ZnONRs/Ni-foam/α-Fe_2_O_3_NPs nanocomposite	CV	10–50 ppb	4.12 ppb	17
Gold nanoparticles	CV	2–12 mM	8 mM	55
Au-CNSm over SPCE	CV	0.0001–100 mM	0.0001 mM	56
AuNPs/SiNPs/SPCE	LSASV	10–100 ppb	5.6 ppb	57
ZnO NRs/α-Fe_2_O_3_ NPs	SWV	10–50 ppb	6.89 ppb	This work
ZnO NRs/α-Fe_2_O_3_ NPs/Au NPs nanocomposite	LSV	10–50 ppb	2.25 ppb	This work

### 3.6 Selectivity and the effects of interfering species

**Fig. 8:**
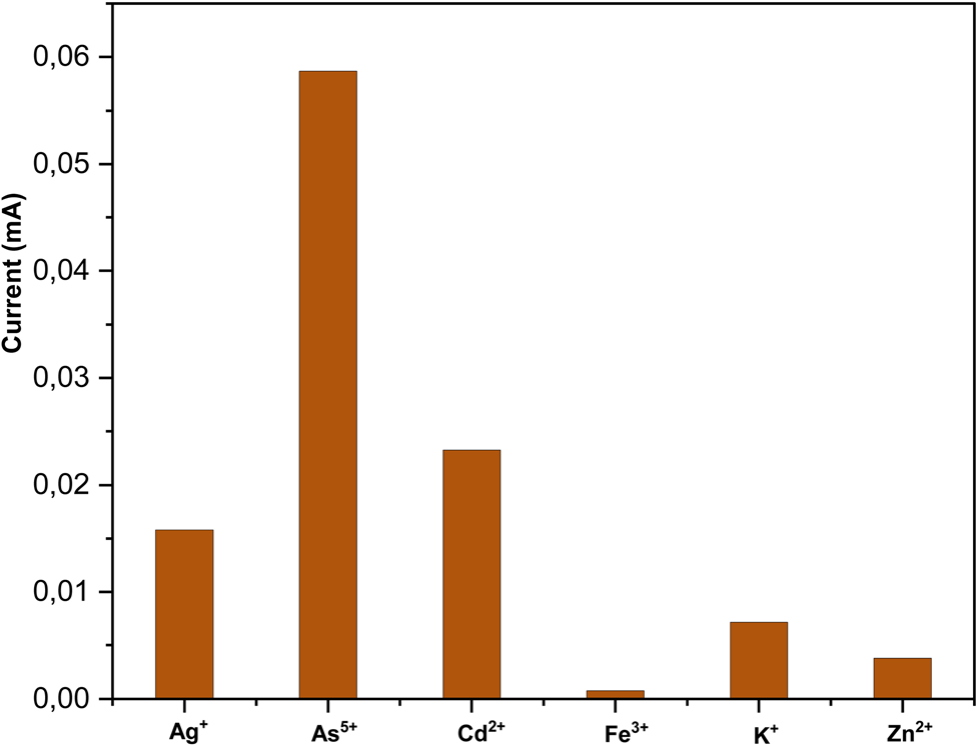
Effect of interference due to the presence of other interfering metal ions under similar experimental conditions (50 ppb) to those used during the detection of arsenic(v) using the ZFA-0.45 mM nanocomposite electrode.

In order to investigate the selectivity of the proposed sensor, which is an important parameter for real aqueous sample applications, we evaluated the selectivity of the fabricated electrodes in the presence of potentially interfering factors, including Ag^+^, Cd^2+^, Fe^3+^, K^+^ and Zn^2+^ under similar experimental conditions to those used for the determination of arsenic(v) in the dispersion of the ZFA-0.45 mM (50 μg L^−1^). To study the interference effect of other metal ions, salts like KCl, AgNO_3_, Cd(NO_3_)_2_·4H_2_O, Zn(NO_3_)_2_·6H_2_O, and Fe(NO_3_)_3_·9H_2_O were prepared and used in the selectivity experiments. The concentration of metal salts was fixed at 50 μg L^−1^ with 1 M KOH as the electrolyte. It was observed that in the presence of metal ions that were 3 times smaller than arsenic(v), there was significant interference, as seen in Fig. 8. The selectivity experiments indicated that the ZFA-0.45 mM electrode can be used successfully for the detection of arsenic(v) since it provides low interference from other metallic ions.

### 3.7 The sensing mechanism of the ZnO NRs/α-Fe_2_O_3_ NPs/Au NPs composite electrode

**Fig. 9:**
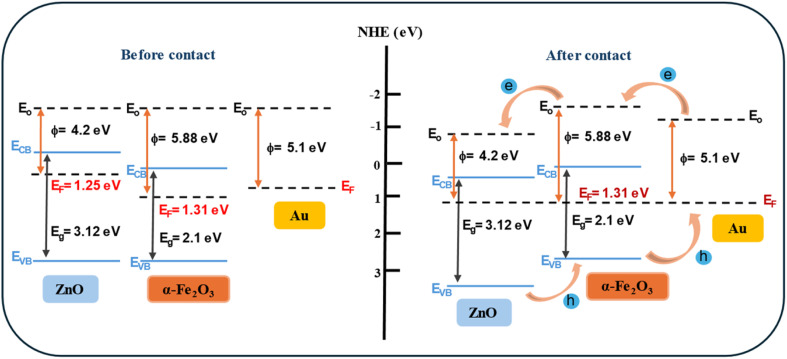
Equilibrium energy band structure diagrams of the ZnO NRs/α-Fe_2_O_3_ NPs/Au NPs composite before and after contact between the ZnO NRs, α-Fe_2_O_3_NPs and Au NPs.

The mechanism of arsenic(v) detection, generation of e^−^/h^+^ pairs, and efficient separation of these charges in the ZFA nanocomposite are proposed, as shown in Fig. 9. The ZFA nanocomposite, conduction band (CB), and valence band (VB) energies of both semiconductors were calculated using Mulliken electronegativity.^58,59^

1 *E*_VB_ = *χ* − *E*_c_ + 0.5*E*_g_

2 *E*_CB_ = *E*_VB_ − *E*_g_

where *χ* is the absolute electronegativity of the semiconductors, *E*_c_ is the energy of a free electron on the hydrogen scale (4.5 eV), *E*_g_ is the energy band gap of semiconductors, *E*_CB_ and *E*_VB_ are the conduction and valence band edge positions. From formulas (1) and (2), we determined that the conduction and valence band energies of the ZNRs were −0.30 eV and 2.82 eV, while the conduction and valence band energies of the α-Fe_2_O_3_ NPs were 0.27 eV and 2.37 eV, respectively. As is well known, ZnO and α-Fe_2_O_3_ are n-type semiconductors with Fermi energy levels lying in the middle of the CB and the VB. After the growth of the ZF, an n–n heterojunction is formed between the ZnO and the α-Fe_2_O_3_ NPs. When Au NPs are deposited onto the surface of the ZF, it is expected to facilitate carrier transfer from the Au NPs to the α-Fe_2_O_3_ NPs and then to ZnO NRs. When ZFA is grown, and due to the energy match between the defect's emission and the SPR, Au NPs could absorb the defect emission energy. In this case, the electrons could efficiently be transferred to a higher energy state. This could lead to the electrons being easily transferred into the conduction bands of α-Fe_2_O_3_ NPs and ZnO NRs. Moreover, deposits of Au NPs on the surface of ZF lead to an efficient charge separation of generated electron–hole pairs in a semiconductor. The Au NPs are very effective traps for electrons due to the formation of a Schottky barrier at the metal–semiconductor contact.^60–62^

## 4. Conclusion

ZFA nanocomposite electrodes were successfully synthesized by the low-temperature hydrothermal chemical and dip-coating methods. The ZF nanocomposite benefits from the synergetic effect and improves the charge separation and transport properties when compared to bare ZNRs. In view of the observed optical properties, the addition of Au NPs on the surface of the ZF further improved the sensing performance due to the effect of SPR from the Au NPs. The Au NPs synthesis conditions were varied to determine the optimum process. It was determined that samples synthesized with 0.45 mM of the Au precursor showed the best performance and were further utilized for arsenic sensing. The electrochemical sensing behavior of the optimized ZFA nanocomposite electrode was investigated *via* linear sweep voltammetry and square wave voltammetry in different solutions with a range of arsenic(v) concentrations from 0 to 50 μg L^−1^. A supporting solution of 1 M KOH was found to be the best for arsenic(v) detection. A reasonable and acceptable lower limit of detection (LOD) was obtained from the calibration study. From the calibration curve, the LOD value was found to be as low as 2.25 ppb, a value that is lower than the highest recommended allowed limit for arsenic in drinking water suggested by the World Health Organization. Our results demonstrate that the ZFA nanocomposite electrode provides superior arsenic sensing compared to the ZF electrode. These findings indicated that the ZFA hybrid configuration can be further developed and utilized to achieve efficient sensors for arsenic(v) detection in drinking water.

## Author contributions

The results achieved in this study were due to the contribution of all authors. In addition, all authors have read and agreed to the published version of the manuscript.

## Conflicts of interest

The authors declare no conflict of interest.

## Supplementary Material

RA-015-D5RA05210F-s001

## Data Availability

All data relevant for the production of the results presented in this work are included within the article and SI. See DOI: https://doi.org/10.1039/d5ra05210f.
