# Supplementary material for: Effect of Au nanoparticles on ZnO nanorods/α-Fe2O3 electrochemical sensor performance
Source: RSC Adv. 2025 Sep 17;15(40):33804–15. doi: 10.1039/d5ra05210f (PMC12441894; doi:10.1039/d5ra05210f)
Supplement: RA-015-D5RA05210F-s001 [file RA-015-D5RA05210F-s001.pdf]

Supplementary Information (SI) for RSC Advances.
This journal is © The Royal Society of Chemistry 2025

Effect of Au nanoparticles on ZnO nanorods/α-Fe_2O_3 electrochemical sensors performance

Sreymean Ngok [1*], Xianjie Liu[2], Magnus Willander[1], Omer Nur [1]

[1] Department of Science and Technology, Physics Electronics and Mathematics, Linköping University, SE-601 74 Norrköping, Sweden

[2] Department of Science and Technology, Laboratory of Organic Electronics, Linköping University, SE-60174, Norrköping, Sweden

Supplement data

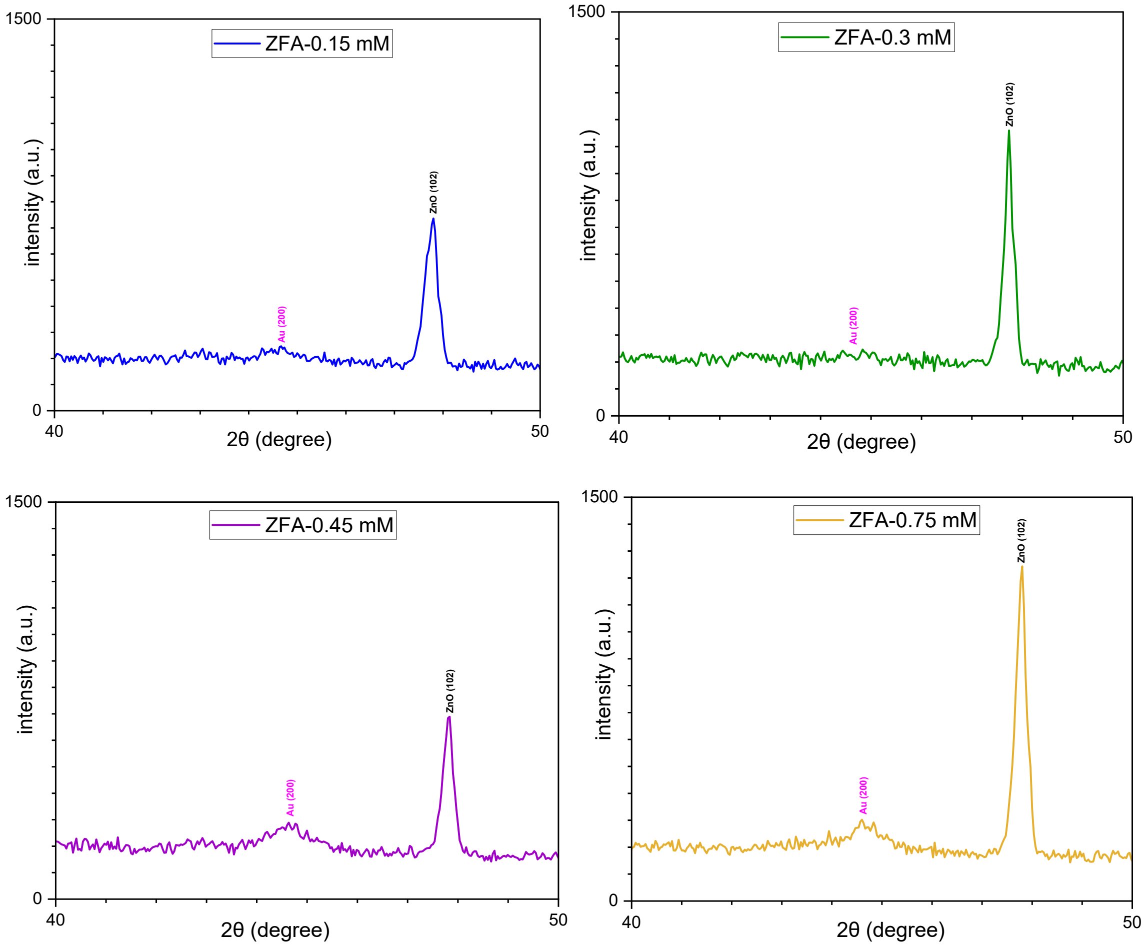

Figure S1: The XRD spectra showing the Au NPs for different Au precursor concentrations as denoted in each spectrum.
